# *Achromobacter* spp. healthcare associated infections in the French West Indies: a longitudinal study from 2006 to 2016

**DOI:** 10.1186/s12879-019-4431-3

**Published:** 2019-09-10

**Authors:** Karine Marion-Sanchez, Karine Pailla, Claude Olive, Xavier Le Coutour, Christian Derancourt

**Affiliations:** 1 Department of Hospital Hygiene, CHU Martinique, Fort-de-France, Martinique; 2 Unité de Surveillance et de Prévention des Infections Nosocomiales, CHU de Martinique, Site Pierre-Zobda-Quitman, CS 90632, 97290 Fort-de-France Cedex, Martinique; 3 Bacteriology Laboratory, CHU Martinique, Fort-de-France, Martinique; 4 Medical School, Université de Caen Normandie, Caen, France; 5 Department of Clinical Research, CHU Martinique, Fort-de-France, Martinique

**Keywords:** *Achromobacter* spp.*,* healthcare associated infection, Immunocompetent, Resistance, retrospective study

## Abstract

**Background:**

Bacteria of the *Achromobacter* genus, more particularly *xylosoxidans* species, are responsible for various healthcare associated infections (HAI) which are increasingly described since the last decade. Cystic fibrosis (CF) patients are considered as potential reservoirs in hospitals. We performed a retrospective study to estimate the frequencies of *Achromobacter* spp. HAI among patients from French West Indies, to determine characteristics of infected patients and establish a possible link between CF and infections.

**Methods:**

All adults with at least one *Achromobacter* spp. positive sample and infection criteria in accordance with European official definitions of HAI, hospitalized in University Hospital of Martinique from 2006 to 2016 for more than 48 h, were included. Patient clinical features, immune status and underlying diseases were obtained from medical files. A list of CF patients was given by clinicians.

Antibiotic-susceptibility profiles of the strains were determined using an automated method.

**Results:**

Mean incidence density was 0.038/1000 days of hospitalization. *Achromobacter* spp. HAI evolved as an endemic situation with a low but pretty much stable incidence rate over the 11-year observation period. An epidemic peak was noticed in 2013. Among the 66 included patients, 56.1% were immunocompetent and no one had CF. Pneumonia and bacteraemia were the two main HAI. Among the 79 isolated strains, 92.4% were resistant to at least 1 major antibiotic and 16.4% met the definition of multidrug-resistant bacteria.

**Conclusions:**

This microorganism, little known in our country because of the scarcity of CF patients, represents a threat for both immunosuppressed and immunocompetent patients and a therapeutic challenge because of its high resistance.

## Background

*Achromobacteria*, multiresistant opportunistic hydro-telluric pathogens, are emerging in hospital environments [1]. The most frequently encountered species is *Achromobacter xylosoxidans* which has been found in various contaminated solutions in hospitals, such as dialysis water, demineralized water, water from nebulizers, humidifiers, incubators, extracorporeal circulation systems, poorly preserved heparin flasks and even antiseptic and disinfectant solutions because of an increasing acquired resistance to these products [2–4]. Its preferential temperature of growth is 30 °C, which makes it particularly adapted to tropical climate.

Research on *Achromobacter* began in the early 1970s, stagnated for 10 years and has increased exponentially over the last decade in parallel with the rise of related HAI [5]. However, clinicians remain poorly informed and more often consider these bacteria as contaminants [6].

*Achromobacter,* most notably *xylosoxidans,* is known to colonize the respiratory tract of CF patients and most authors reported its increased prevalence in that population [7–9], who might represent the microorganism’s main human reservoir in healthcare facilities where cross-contaminations have been described [8, 9]. Various *Achromobacter* HAI have been reported, especially in ophthalmology [10], pulmonology [1, 6], surgery [11–13] mainly documented by case reports [3, 11–17] or clustered cases of infections during small outbreaks [18–20]. But, to our knowledge, only three retrospective studies are available and they all focused on targeted pathologies: two evaluated respectively 54 and 13 bacteraemia in a 10 -year observational period in Spain [21, 22] and one concerned 41 pneumonia in the elderly in China [6]. None gave a detailed inventory of the different types of HAI.

Infections related to these bacteria are considered mainly associated to an immunocompromised status [3, 14, 18, 20]. However, numerous cases of *Achromobacter* infection occurring in immunocompetent patients have been published [11, 12, 15, 16].

The main objective of our study was to estimate frequencies and distribution of *Achromobacter* spp. HAI among all adults hospitalized in the University Hospital of Martinique between 2006 and 2016. The secondary goals were to describe characteristics of infected patients, particularly their immune status, and to establish a possible link between such infections and CF.

## Methods

### Design

We performed a retrospective monocenter study from January 2006 to December 2016 based on original data extracted from our Bacteriology Laboratory database (SIRweb™, i2a, Montpellier, France) in a listing of all *Achromobacter* spp. positive clinical samples analyzed.

The study was conducted in the University Hospital of Martinique, a 1484-bed public facility resulting from the merger of 7 sites. Our team of 3 physician-hygienists and 5 nurse-hygienists monitors HAI throughout the whole institution.

### Study population

We included all adults (> 18 years old) hospitalized between January 1st 2006 and December 31th 2016 in the largest site of our institution, Pierre-Zobda-Quitman1 Hospital, including 35 medical and surgical units (570 beds).

Inclusion criteria were patients with ≥1 clinical isolate positive for *Achromobacter* spp. with associated clinical, radiological, biological criteria in accordance with European Union case definitions [23], regardless of the type of infection. Samples were taken into account only if they were qualitatively and quantitatively consistent with the microbiological criteria [23] and if the infection was confirmed by clinicians. Only patients with HAI were included. Cases were considered as HAI when infection occurred more than 48 h after admission, as defined by the European Union [23]. Conversely, patients whose infections occurred less than 48 h post-admission, considered as community cases, were excluded from the study, as well as positive samples corresponding to colonizations.

Duplicates, i.e. strains with the same antimicrobial susceptibility isolated from the same sample type from a given patient, were excluded.

### Data sources

Data collected concerned basic demographics (sex, age), patient stay (admission date, unit), sample characteristics (specimen type, time of positive culture, concomitant microorganisms (same specimen), and antibiotic susceptibility), HAI criteria and validation by clinician, iatrogenic causes of immune deficiency (immunosuppressants, chemotherapy, radiotherapy, organ transplantation, glucocorticoids), CF and/or other relevant underlying or autoimmune diseases, presence of invasive medical device (intra-vascular devices, prothesis, implants, intubation, urinary tract catheter …). Ventilator-associated pneumonia and catheter-related bacteraemia were defined according to the European Union criteria [23].

Demographics, patient stay and sample data were extracted from SIRweb™. Financial Services provided the number of admissions and patient-bed days.

Clinical, biological and radiological criteria of *Achromobacter* spp. HAI and underlying diseases were obtained from consulting medical files or from a coding software for medical procedures linked to hospitalization and operative reports (CORA software-Maincare Solutions, Cestas, France).

A list of CF patients followed from 2006 to 2016 was provided by the clinicians from the Department of Pulmonology.

Patients meeting one of the following criteria described in our Nosocomial Infections National Prevalence Survey (NINPS) protocol [24] were considered immunocompromised: i.e., solid tumor or hemopathy, organ transplantation, radiotherapy, chemotherapy, immunosuppressive therapy, high-dose prednisolone (> 5 mg/kg/day) or prolonged (> 30 days) corticosteroid use or Human Immunodeficiency Virus infection with < 500 CD4^+^ cells/mm^3^. The immunocompromised status was established from the following data: history of organ transplantation (provided by clinicians), chemotherapy, radiotherapy or immunosuppressive therapy from 2006 to 2016 (provided by pharmacists and Medical Information Department), and the existence of co-morbidities requiring long-term general corticosteroid therapy (provided by CORA software).

### Microorganisms and antibiotic susceptibility

Methods remained consistent along the 11-year period of the study. Strains isolated from biological samples were first identified with the API 20NE system (bioMerieux, Marcy-l’Etoile, France). Confirmation was performed later by MALDI-TOF (Microflex LT, Brüker, Germany) on the preserved strains. One strain was identified using nrdA sequencing [25].

Antibiotic-susceptibility profiles were determined using the automated Microscan Walkaway® system (Beckman Coulter, Villepinte, France) with NEG Urine combo panel type 57 micro-plates. Briefly, a micro-inoculum taken from a bacterial colony using the Prompt Inoculation System-D was suspended in saline buffer (0.5 McFarland Standard). The suspension was used to inoculate a 96-well plate coated with standard biochemical indicators and antibiotics. The plate was incubated at 37 °C for 24 h and bacterial growth was determined by measuring the optical density at 600 nm. This technique enables bacterial identification and minimal inhibitory concentration (MIC) calculation. Six major antibiotics were tested (i.e., main molecules usually used to treat *Achromobacter* infections) [3, 8]. They are cited in Table 4.

Resistant strains were defined as strains able to become resistant to one or several antimicrobial agents used for therapy or prophylaxis, according to the European Center for Diseases prevention and Control [26]. Multidrug-resistant (MDR) strains were defined as non-susceptible to at least one agent in > 3 different antimicrobial categories, according to the International Expert Proposal and considering data from *Pseudomonas aeruginosa* [27].

Strains exhibiting intermediate or strong resistance to the tested molecule were considered resistant, as defined in the NINPS protocol [24]. Resistance rate for one given antibiotic was calculated as follows: ((total number of isolated strains - susceptible strains) / total number of isolated strains) X 100, as described in our National Surveillance of Antibiotic Consumption protocol [28].

### Statistical analyses

Incidence was defined as number of new cases during a period/number of hospitalized patients during the same period. Incidence density was defined as number of new cases during a period/number of days of hospitalization during the same period.

Categorical data, expressed as numbers and percentages, were compared with χ^2^ or Fisher’s exact test, and quantitative data, expressed as means +/− standard deviation (SD), were compared using Student’s t-test. Analysis was performed using SAS /STAT software (SAS, France). Significance was defined as *p* < 0.05.

## Results

### Study population

The SIRweb™ database initially yielded 107 *Achromobacter* spp-positive clinical isolates. All were identified as *Achromobacter xylosoxidans*.

**Fig. 1:**
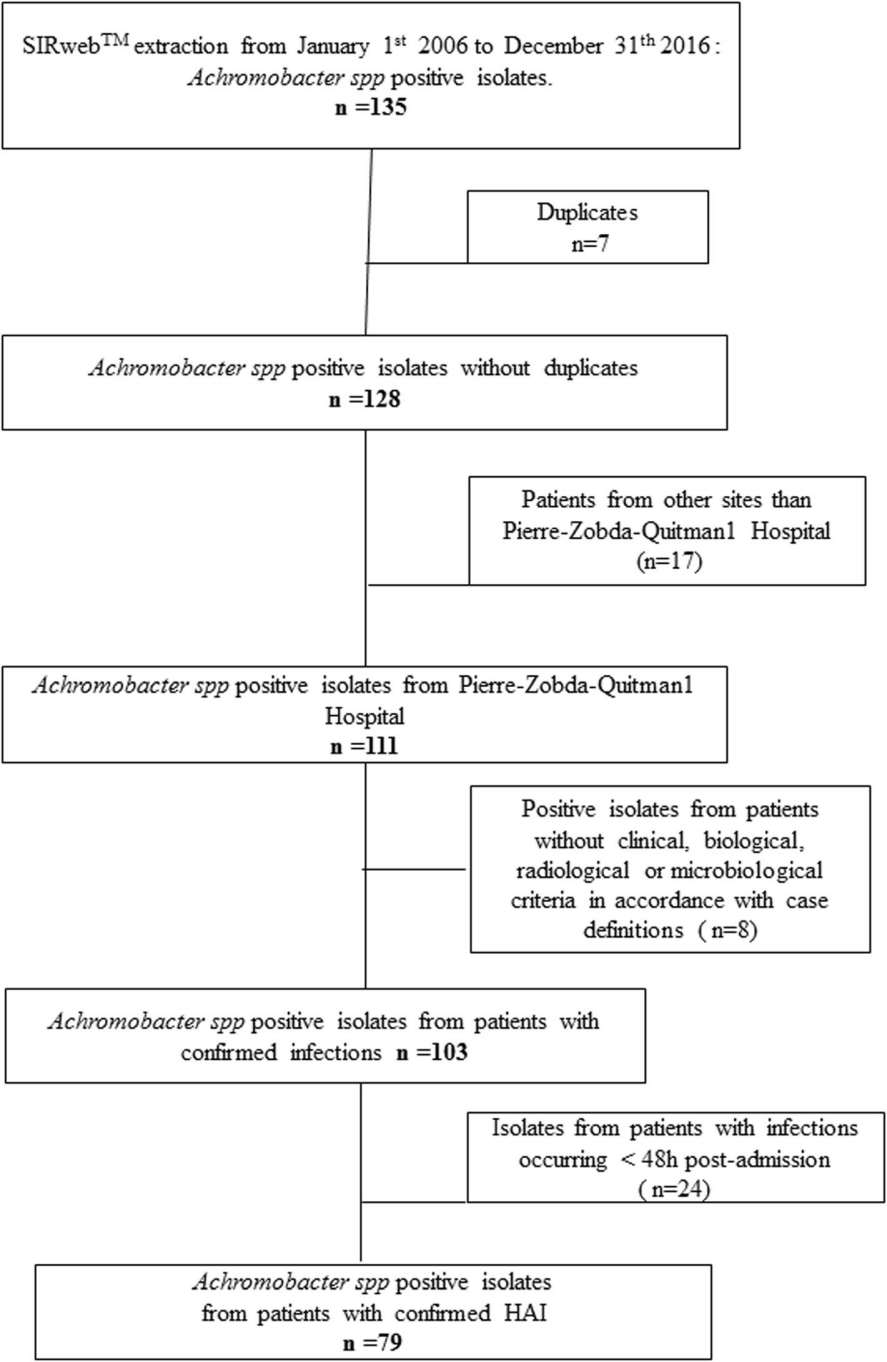
Study flow chart. 135 positive samples extracted from laboratory data base; application of exclusion criteria; 79 resulting included cases = 69 HAI = 66 patients

After applying inclusion and exclusion criteria, our dataset included 79 positive clinical isolates, corresponding to 66 patients and 69 HAI (one patient may have more than one positive clinical isolate and may develop more than one HAI). Details are given by the study flow chart (Fig. 1).

Basic demographics are shown in Table 1. Patients with *Achromobacter* spp. HAI were predominantly men. Their mean age was 61.5 +/− 17.7 years, with 53% of them < 65 years old, but women were 10.8 years older than men (*p* = 0.01).

**Table 1:** Demographics and clinical features of 66 the patients with *Achromobacter* spp. HAI

Criteria	Value n (%)
Sex	
Male	42 (63.6)
Female	24 (36.4)
Age in years (mean ± SD)	61.5 ± 17.7
> 65 years	31 (47)
Days to diagnosis (mean ± SD)	23 ± 20
Hospitalization unit	
Medicine	28 (42)
Intensive care	20 (30.3)
Surgery	18 (27.3)
Immune status	
Immunocompromised	29 (43.9)
Immunocompetent	37 (56.1)
Underlying disease	
Cardiovascular	16 (24.2)
Solid tumor	15 (22.8)
Digestive	8 (12.1)
Malignant hemopathy	7 (10.6)
Respiratory	7 (10.6)
Others	7 (10.6)
Neurological	6 (9.1)
Invasive medical devices	55 (83.3)
Cystic fibrosis	0

Results are expressed as n (%) or mean +/− standard deviation (SD).

### Frequencies

The mean annual incidence (pooled mean of all cases) was 1.45/10,000 patients (95% confidence interval (CI) ± 0.52). The mean incidence density (pooled mean of all cases) was 0.038/1000 days of hospitalization (95% CI ± 0.011).

**Fig. 2:**
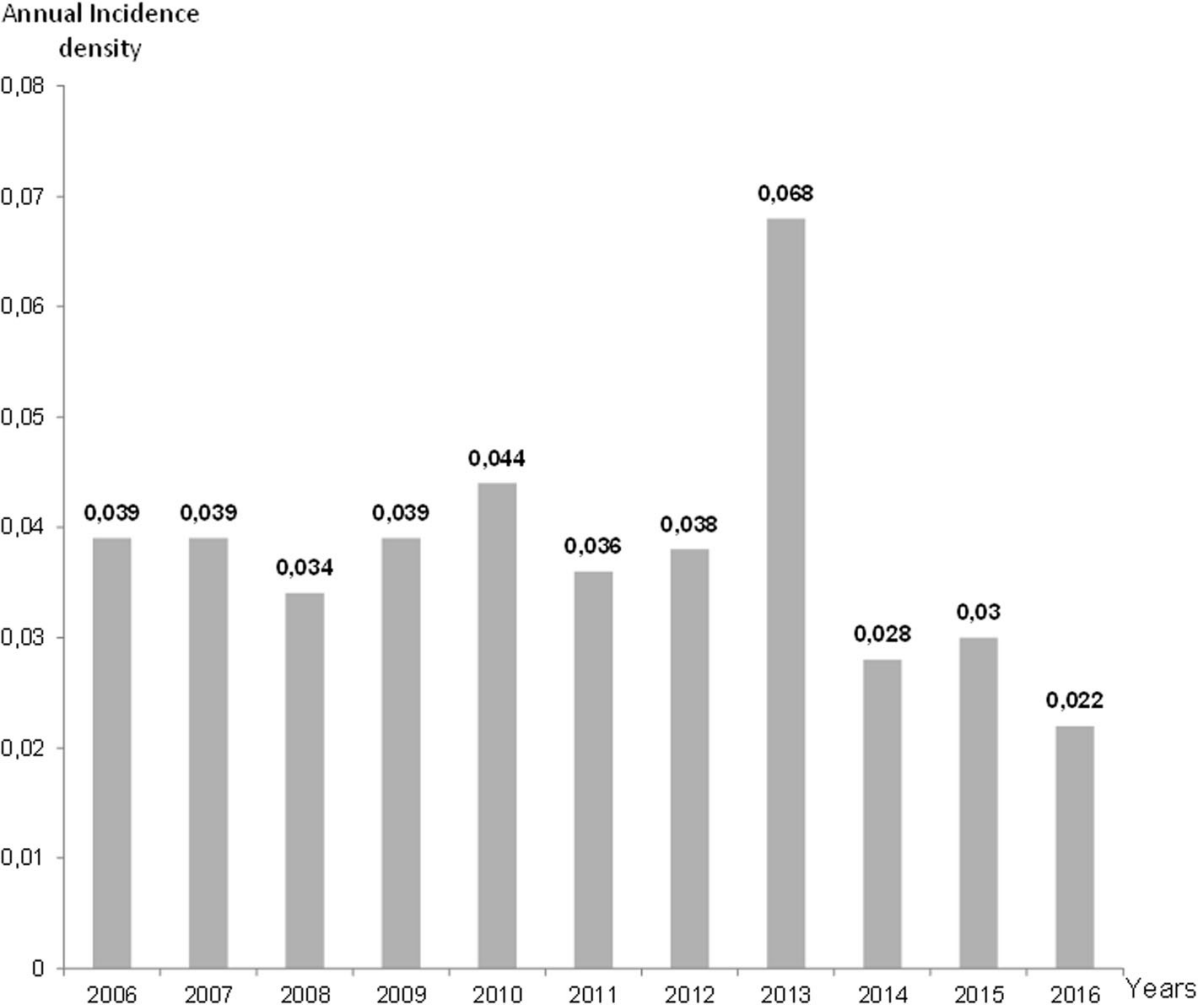
Time-line distribution of annual incidence densities. Annual incidence densities = number of new cases during the year/number of days of hospitalization during the same year

Figure 2 describes the time-line distribution of annual incidence densities. A global stability of annual incidence densities was observed, with an epidemic peak in 2013. It showed a tendency to decrease over the last 3 years.

**Fig. 3:**
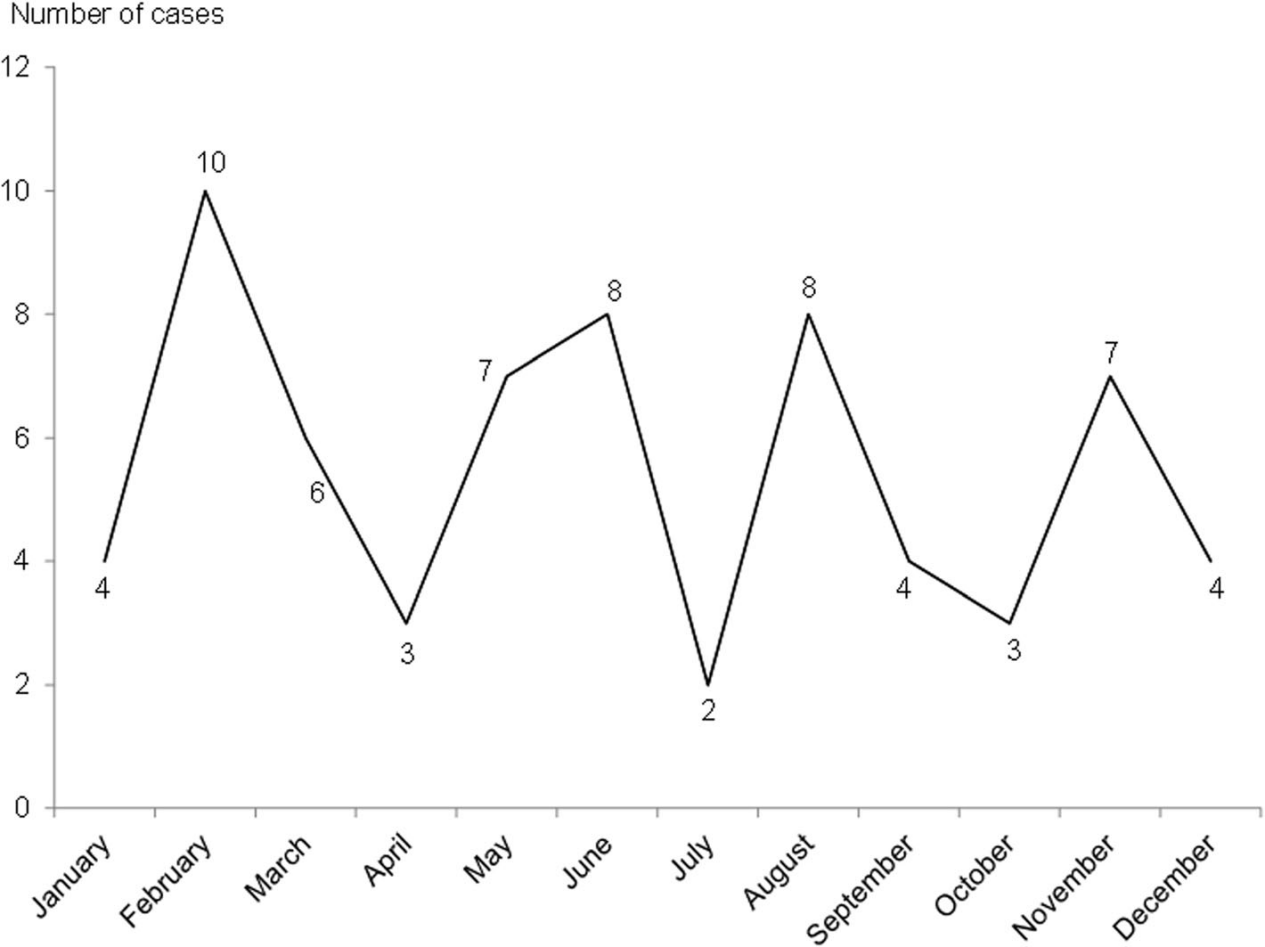
Monthly distribution of included cases (pooled data). 66 included cases from 2006 to 2016 pooled and expressed as number of cases per month of diagnosis

The 66 included cases were pooled in order to study a possible seasonal variability. The monthly distribution of included cases is represented by Fig. 3. The highest number of cases was observed in February, with two other maximum in May–June and August. On the other hand, the same number of cases were diagnosed whatever the season, 34 cases during dry season (November to April, temperature around 27 °C humidity around 65% and little rain) and 32 during cyclonic season (May to October, temperatures around 31 °C humidity up to 80% and heavy rainfall).

### Patients’ clinical features and infections

Patients’ clinical features are described in Table 1. Table 2 gives the distribution of the 69 HAI. HAI were diagnosed within an average of 23 days, with extreme values ranging from 4 to 94 days. Most HAI occurred in medical units, the most common being pneumonia (37.7%) and bacteraemia (33.3%).

**Table 2:** Distribution of the 69 HAI

Infections (*n* = 69)	Frequencies n (%)
Pneumonia	26 (37.7)
Bacteraemia	23 (33.3)
Intra-abdominal infection	7 (10.1)
Urinary tract infection	7 (10.1)
Mediastinitis	3 (4.4)
Upper respiratory tract infection (pharyngitis)	2 (2.9)
Wound infection	1 (1.5)

The bacteriological diagnosis of *Achromobacter* spp. pneumonia, in addition to the clinical, biological and radiological criteria, was based on positive quantitative culture of distal protective aspirates (*n* = 11), broncho-alveolar lavage (*n* = 12) and endotracheal aspirates (*n* = 3). According to the European Union classification, 23 cases were defined as PN1 and 3 as PN2 (i.e. certain (PN1) or probable (PN2) pneumonias with clinical and radiological elements, associated with microbiological documentation).

Among the 66 patients included, 36 had mechanical ventilation at least 48 h before the diagnosis. *Achromobacter* spp. pneumonia acquisition was very significantly higher among those patients than among patients without mechanical ventilation, who developed more particularly other types of *Achromobacter* spp. HAI (χ^2^, *p* = 0.0002). Cardiovascular pathologies were the main underlying diseases among patients with pneumonia (*n* = 8).

All patients with *Achromobacter* spp. bacteraemia presented clinical signs of sepsis. 60.8% (*n* = 14) had catheter-related infections. Other sources were surgical site infections (*n* = 4), and urinary tract infection (*n* = 1). Four patients had no identifiable source of bacteraemia. Cancers (solid tumor + malignant hemopathy) (*n* = 12) and cardiovascular pathologies (*n* = 6) were the main underlying diseases among patients with bacteraemia.

Intra-abdominal infections were peritonitis (*n* = 5) and hepatic abscesses following surgery (*n* = 2). All were diagnosed on positive cultures of purulent material from intra-abdominal space obtained during surgical revision (*n* = 2) or liquid puncture (*n* = 5).

All urinary tract infections were microbiologically confirmed symptomatic infections.

Among the 69 *Achromobacter* spp. HAI, 6 occurred after surgery and met the definition of surgical site infections. One was superficial (wound infection) and 5 were deep infections (3 mediastinitis and 2 deep abscesses).

NINPS criteria for immunocompromised status were met by only 43.9% of the patients. 33.3% of included patients had cancer.

None of the patients had CF, both among included patients and among patients having a simple colonization, excluded from the study.

### Microorganisms and antibiotic susceptibility

Another microorganism was isolated from 24.1% of the 79 samples as described in Table 3.

**Table 3:** Co-infecting pathogens and resistant rates of the 79 clinical isolates

Strains (*n* = 79)	Frequencies n (%)
1 Co-infecting pathogen	19 (24.1)
2 Co-infecting pathogens	6 (7.6)
Resistant strains	73 (92.4)
MDR strains	17 (21.5)

Ten pneumonia (38%), 4 bacteraemia (17%), 4 intra-abdominal infections (57%) and 1 mediastinitis (33%) were polymicrobial. The most frequent co-infecting microbes were *Pseudomonas aeruginosa* (23%), *Klebsiella pneumoniae* (16%), *Candida albicans* (16%), *Stenotrophomonas maltophilia* (13%) and *Staphylococcus aureus* (10%). *P. aeruginosa* and *C. albicans* were mainly found in lower respiratory tract specimens, while *K. pneumoniae* could be isolated from all types of *Achromobacter spp-*positive samples.

Antibiotic-susceptibility profiles of *Achromobacter* spp. isolates are reported in Table 4.

**Table 4:** Antibiotic-susceptibility profiles of the 79 isolated strains

Antibiotic	Antibiotic-susceptibility profile				
	Sensitive n (%)	Intermediate n (%)	Resistant n (%)	Not determined n (%)	Resistance rates (%)
Ceftazidime	62 (78.5)	7 (8.9)	10 (12.7)	–	21.5
Trimethoprim + Sulfamethoxazole	72 (91.1)	6 (7.6)	1 (1.3)	–	8.8
Piperacillin + Tazobactam	59 (74.7)	13 (16.5)	5 (6.3)	2 (2.5)	22.8
Imipenem	66 (83.5)	5 (6.3)	8 (10.1)	–	16.5
Ciprofloxacin	10 (12.7)	22 (37.8)	47 (59.5)	–	87.3
Ticarcillin+ Clavulanic acid	58 (73.4)	6 (7.6)	5 (6.3)	10 (12.7)	13.9

Strains were more susceptible to Trimethoprim + Sulfamethoxazole, Ticarcillin + Clavulanic acid and Imipenem.

Seventy three out of the 79 isolated strains (92.4%) were resistant to one or more major antibiotics (Table 3). Only 6 strains were susceptible to all tested molecules and 13 strains (16.4%) met the definition of MDR strains, including 3 blood samples (13%). Among those 13 MDR strains, a lower respiratory sample from a 39-year-old immunocompromised woman with acquired immunodeficiency syndrome yielded a strain resistant to all antibiotics tested.

All the 6 susceptible strains were isolated in patients hospitalised before 2012 and most MDR were isolated after 2013 (*n* = 9), suggesting an increase in the resistance of the strains during the observation period.

## Discussion

Our study showed that an average of 1.15/10,000 patients a year has developed *Achromobacter* spp. HAI in our institution from 2006 to 2016. Those infections, especially pneumonia or bacteraemia, appeared within an average 23-days hospitalization period.

Infected patients were predominantly men, relatively young, with cardiovascular pathologies and mostly immunocompetent. These *Achromobacter* spp. HAI were not related to CF, none of our included patient being affected by this pathology.

In all isolates analysed, only one and the same species, *Achromobacter xylosoxydans*, was identified. However, the methods used for identification were not the recommended method to distinguish species, i.e., nrd A sequencing [29], so, all isolates were referred as *Achromobacter* spp.

Since 2011, our institution regularly conducts an annual national surveillance of HAI in intensive care unit [30]. The mean annual incidence over the last 7 years is 20/100 patients (including all infections and all microorganisms). Ventilator associated pneumonia (VAP) and catheter-related bacteraemia are the most common infections and *Klebsiella pneumoniae* is the most frequently encountered bacteria.

In our study, although the annual incidence of *Achromobacter* spp. HAI was fairly low, it remained pretty much stable over the 11-year observation period, meaning that *Achromobacter* spp. HAI evolved as an endemic situation. Only one epidemic peak was observed in 2013. That year, our institution was merged with 2 other sites to create the University Hospital of Martinique. Well known organizational disorders generated by this event may probably explain a global and transient loosening of good hygiene practices. When compared to the results of our annual surveillance in intensive care unit [30], we note stable infection rates (26.4% in 2012, 26.6% in 2013) but an increase in *Pseudomonas aeruginosa* infections (22.6% of total isolated germs in 2013 versus 4.8% in 2012)*.* The merger had a low impact on the activity and behaviour of healthcare workers in intensive care unit because they were already used to receive patients from all other sites, but it is likely that the loosening of good hygiene practices mainly concerned environment management in the whole institution. Despite a slight decrease in incidence of *Achromobacter* spp. HAI at the end of the study, we observed an increase in 2017 (0.037/1000 days of hospitalization).

Half of the cases in this study were diagnosed in February, May–June and August (*n* = 33). Surprisingly, these months represent vacation periods, with carnival days in February, a succession of public holydays in May and June and summer holidays in August. The lack of staff or the use of substitute staff during these periods may explain once again a loosening of good hygiene practices. Contrary to what we might think, *Achromobacter* spp. HAI seem to be linked more to human behavior than to climatic conditions. In fact, no seasonal variation could be demonstrated. A recent retrospective study on *Achromobacter xylosoxidans* colonizations in young children with CF contradicts our observations, showing a seasonal variation in acquisition of the bacteria, with a peak incidence in the winter. The authors explain it by higher prevalence of viral respiratory infections in the winter increasing airway susceptibility to bacterial colonization. However, it is the only study available dealing with seasonality and *Achromobacter*, in a context very far from our own in terms of population, pathologies and climate [31].

Because of the low incidence of *Achromobacter* spp. HAI, we decided to undertake a retrospective study. Knowing that susceptibility to infections, response to antibiotics, clinical features and outcomes differ between children and adults, we considered only adult cases. The availability of data extracted from SIRweb™ software set the inclusion period at 11 years, which identified only 66 patients, a relatively small population. Analyses of different variables generated small subgroups among them, making statistical comparisons difficult if not impossible.

Old age (> 65 years) does not seem to favor infections in the studied population. Indeed, average age was similar to that of all hospitalized patients (61.9 years in 2016) and did not exceed 65 years. Because most studies targeted specific populations (newborns, elderly …), available published information is insufficient to compare and/or confirm our results.

One of the main findings of our study is that there is no link between *Achromobacter* spp. HAI and CF patients. Because of ethnic specificities, CF is very scarce in Martinique. Only 39 patients were followed for CF from 2006 to 2016. Among these patients, none was included in our study and none was even colonized by *Achromobacter* spp. While prevalence of *Achromobacter* spp. in CF patients is 6.3% in Metropolitan France [8], it is 0% in Martinique. So, such patients cannot be a potential human *Achromobacter* spp. reservoir in our institution. Our series of 26 *Achromobacter* spp. pneumonias without CF completes published series (32 cases) [1], these 26 cases alone accounting for 45% of total cases published over 40 years.

According to our results, pneumonia represented the main *Achromobacter* spp. HAI among our patients over the past 11 years in Martinique and those infections do not seem to affect preferentially patients with cancer. Our findings contrast with Swenson and Sadikot’s observations showing a strong relationship between hematological or solid-organ malignancies and *Achromobacter* spp. pneumonia [1]. Conversely, our results are in accordance with Liu et al who described 41 cases of *Achromobacter xylosoxidans* pneumonia in the elderly. Only 19.5% had malignancies and cardiovascular pathologies were the main underlying disease [6].

The mechanical ventilation–pneumonia association we found is not specific to *Achromobacter* spp. HAI. In fact, mechanical ventilation is the major cause of nosocomial pneumonia [32] and concerns 87.4% of cases in France [30]. The high rate of ventilator-associated pneumonia may explain the polymicrobial character of a great number of lower respiratory tract samples from our study. As recently described by Rodrigues et al, bacterial and/or fungal biofilms, most of them being polymicrobial, develop on the surface of endotracheal tubes and represent the starting point for most VAP [33].

Such mechanical ventilation association confirmed the nosocomial character of pneumonia in our study and revived the question about *Achromobacter* spp. digestive carriage, which remains controversial [7, 11, 14, 34]. Although the gastro-pulmonary route is not considered to be the major route for the development of VAP, gastric colonization remains a potential endogenous source of infection [32]. In fact, a recent study even showed a relationship between digestive tract colonization related to extended-spectrum beta-lactamase producing Enterobacteriaceae (ESBLE) and the occurrence of ESBLE VAP [35]. *Achromobacter* spp. digestive colonization may occur after ingestion of contaminated drinking water or during ocean/river activities that are very common in Martinique. Moreover, in our study, intra-abdominal pathologies represented both the third underlying disease and the third HAI. We plan to complete this study by identifying natural *Achromobacter* spp. sources in our environment, especially in rivers, Caribbean Sea or Atlantic Ocean, and by examining potential digestive carriage using rectal swabs.

The second most common *Achromobacter* spp. HAI in our study was bacteraemia, most being related to catheter. Our 23 cases complete the 13 cases observed by Perez-Barrangan et al in their recent 10-years retrospective study, which was conducted in Spain during the same period [22]. Predominance of catheter-related infections appears more clearly in our study (60.8% versus 30.7%). Both studies highlight similar most common underlying diseases and similar rates of polymicrobial bacteraemia (15 and 17%). Immunocompromised rates could not be compared because it was not clearly determined in the Spanish study. Conversely, the same MDR definition was used and the Spanish isolates seem to show a highest resistance compared to the Martinican ones (38.4% versus 13%).

One of the main features of our population was their low immunocompromised rate. Compared to the literature [3, 14, 18, 20] more than 50% of our patients did not meet the immunosuppression criteria [23]. *Achromobacter* spp. is classically considered as an opportunistic pathogen, theoretically described as microorganisms mainly infecting immunocompromised individuals. Our findings could question that hypothesis. Moreover, a recent publication defines for the first time both immunosuppressed and immunocompetent populations as potential targets for this bacterium [36].

Larger multicenter studies are needed to enable multivariable analyses to assess potential associations between *Achromobacter* spp. and the immunocompromised rate.

## Conclusions

In Metropolitan France, genus *Achromobacter* is emerging among CF patients and among immunocompromised patients. In Martinique, it evolves as an endemic situation and seems to affect mainly immunocompetent patients without CF. It appears as neither an innocuous environmental bacterium nor a simple contaminant. It may be a future threat for patient safety in the hospital environment. In vitro experiments and environmental investigations are currently underway in our institution to highlight potential sources of contamination and their portals of entry.

## Data Availability

The datasets used and analyzed during the current study are available from the corresponding author on reasonable request.

## Abbreviations

CF: Cystic Fibrosis
CI: Confidence Interval
ECDC: European Center for Disease prevention and Control
ESBLE: Extended-Spectrum Beta-Lactamase producing Enterobacteriaceae
HAI: Healthcare Associated Infections
MDR: Multi-Drug Resistant
MIC: Minimal Inhibitory Concentration
NINPS: Nosocomial Infections National Prevalence Survey
SD: Standard Deviation
VAP: Ventilator-Associated Pneumonia
